# Genetic control of adult neurogenesis: interplay of differentiation, proliferation and survival modulates new neurons function, and memory circuits

**DOI:** 10.3389/fncel.2013.00059

**Published:** 2013-05-14

**Authors:** Felice Tirone, Stefano Farioli-Vecchioli, Laura Micheli, Manuela Ceccarelli, Luca Leonardi

**Affiliations:** Institute of Cell Biology and Neurobiology, National Research Council, Fondazione Santa LuciaRome, Italy

**Keywords:** adult neurogenesis, differentiation, proliferation, Btg1, Btg2, Tis21, hippocampus, memory

## Abstract

Within the hippocampal circuitry, the basic function of the dentate gyrus is to transform the memory input coming from the enthorinal cortex into sparse and categorized outputs to CA3, in this way separating related memory information. New neurons generated in the dentate gyrus during adulthood appear to facilitate this process, allowing a better separation between closely spaced memories (pattern separation). The evidence underlying this model has been gathered essentially by ablating the newly adult-generated neurons. This approach, however, does not allow monitoring of the integration of new neurons into memory circuits and is likely to set in motion compensatory circuits, possibly leading to an underestimation of the role of new neurons. Here we review the background of the basic function of the hippocampus and of the known properties of new adult-generated neurons. In this context, we analyze the cognitive performance in mouse models generated by us and others, with modified expression of the genes Btg2 (PC3/Tis21), Btg1, Pten, BMP4, etc., where new neurons underwent a change in their differentiation rate or a partial decrease of their proliferation or survival rate rather than ablation. The effects of these modifications are equal or greater than full ablation, suggesting that the architecture of circuits, as it unfolds from the interaction between existing and new neurons, can have a greater functional impact than the sheer number of new neurons. We propose a model which attempts to measure and correlate the set of cellular changes in the process of neurogenesis with the memory function.

The adult brain contains neurogenic niches in the subventricular zone (SVZ; Doetsch et al., 1999; Alvarez-Buylla and Lim, 2004), which is adjacent to the lateral ventricle, and in the dentate gyrus of the hippocampus (Cameron et al., 1993; Kuhn et al., 1996; Kempermann et al., 1997b), where neural stem/progenitor cells (NSPs) continuously generate post-mitotic neurons of different types. The process of adult hippocampal neurogenesis from astrocytic putative neural stem cells (Seri et al., 2001) has been divided into six developmental stages (Kempermann et al., 2004), in which putative neural stem cells (named type-1 cells) develop into post-mitotic neurons through three consecutive stages of progenitor cells (type-2ab and type-3 cells; Filippov et al., 2003; Fukuda et al., 2003; Kronenberg et al., 2003).

The basic function assigned to the dentate gyrus circuitry consists in the ability to disambiguate related memory stimuli (Rolls and Kesner, 2006; see below). In this context, new adult-generated neurons in the dentate gyrus appear to be required for hippocampus-dependent learning and memory (Bruel-Jungerman et al., 2007; Zhao et al., 2008) by providing pattern separation, a type of neuronal plasticity that facilitates the acquisition and separation of closely spaced memories (Clelland et al., 2009; Aimone et al., 2011; Sahay et al., 2011; Nakashiba et al., 2012).

Notably, the vast majority of studies on the functional role of new neurons have involved either a decrease of their number, by inducing their death (Shors et al., 2001, 2002; Snyder et al., 2005; Saxe et al., 2006, 2007), or an increase of their number through learning and/or physical exercise (Kempermann et al., 1997a; van Praag et al., 1999; Ambrogini et al., 2000; Döbrössy et al., 2003). However, ablation of new neurons did not always give consistent effects on hippocampus-dependent learning tasks (Shors et al., 2001; Madsen et al., 2003; Raber et al., 2004; Snyder et al., 2005). Ablation also makes it impossible to analyze the integration of new neurons into memory circuits.

In this review we summarize the framework of the basic function of the hippocampus and of the putative role of new neurons. In this context, we also attempt to analyze the functional aspects of new adult-generated dentate gyrus neurons through a different perspective, i.e., without ablating them, but by modulating their differentiation.

## Basic function of the dentate gyrus

The hippocampus is composed of the so called tri-synaptic circuit, comprising the projections directed from the entorhinal cortex layer 2 via the perforant path to granule neurons in the dentate gyrus. This in turn projects to CA3 through the mossy fibers and the projections from CA3 to CA1 through the Schaffer collaterals. The entorhinal cortex is the major input and output system whereby the hippocampus communicates with the rest of the brain, via projections to the dentate gyrus through the perforant pathway and through projections from CA1 to the entorhinal cortex (subiculum; Amaral and Witter, 1995).

A large body of experimental evidence in animal models and also in human patients with hippocampal lesions has indicated an impairment of memories that integrate contextual, spatial or temporal information (for review, see Frankland and Bontempi, 2005).

Several models of consolidation of memory by the hippocampus have been proposed over the years, from the first model of Marr proposing that the hippocampus works as a temporary memory repository before final storage in the cortex (Marr, 1971), to the multiple trace theory suggesting that the hippocampus is always required for rich contextual or spatial detail (see for review Nadel and Moscovitch, 1997; Becker, 2005; Frankland and Bontempi, 2005; Moscovitch et al., 2005; Rolls and Kesner, 2006).

The peculiar integration exerted by the hippocampus on spatial/contextual memories is thought to lie in the ability of the dentate gyrus to remove redundancy in the memory input from the entorhinal cortex, by a process that transforms memories into a sparse and categorized set of outputs to CA3 (Rolls and Kesner, 2006). This is thought to occur as a result of competitive learning, where a role could be played by the non-linearity of the NMDA receptor on synapses of dentate gyrus neurons and the low number of connections of dentate gyrus neurons on CA3 neurons (Rolls and Kesner, 2006). In fact, dentate gyrus granule neurons outnumber those of the entorhinal cortex (in the rat 1,000,000 vs. 300,000; Boss et al., 1985; Rapp et al., 2002) and in the rat each CA3 cell receives approximately 50 mossy fiber inputs, so that the sparseness of this connectivity is high (0.005%; Rolls and Kesner, 2006). Moreover, the granule cell population has in itself sparse activity as only about 2% of granule cells are active during explorative behavior (Chawla et al., 2005). Such a sparse and distributed representation lowers the probability that two different input patterns will overlap, thereby facilitating the process of pattern separation. Additionally, the input from each granule cell is conveyed with high efficiency to CA3 as synapses between granule cells and pyramidal cells in CA3 are very strong, so that a single granule cell is able to activate the firing of CA3 cells, which is further amplified by CA3 collaterals (Henze et al., 2002; Ishizuka et al., 1990).

This circuitry thus suggests that similar input patterns from the entorhinal cortex can be effortlessly transformed by granule neurons of the dentate gyrus into separate representations in CA3, thus favoring the encoding and retrieval of inputs incoming from the cortex as separate (Treves et al., 2008; Rolls, 2010). Very interestingly, there is evidence for a second mechanism able to completely orthogonalize the firing of cells in the entorhinal cortex onto CA3. In fact, in the case of more substantial changes, pattern separation appears to occur on CA3 independently from the dentate gyrus, being probably triggered by direct projections from entorhinal cells to CA3 (Leutgeb et al., 2007).

The whole model referred to the ability of pattern separation exerted by the dentate gyrus may in theory remain substantially similar if new adult-generated neurons are taken into consideration. However, new neurons are more readily activated and have additional properties, as summarized in the following section.

## Properties and function of young neurons in the adult dentate gyrus

Newborn neurons are functionally integrated into the existing dentate gyrus circuitry within 3 weeks, extending their axons to CA3, as indicated by morphological and electrophysiological studies (Hastings and Gould, 1999; Wang et al., 2000; van Praag et al., 2002; Ambrogini et al., 2004; Overstreet et al., 2004; Schmidt-Hieber et al., 2004; Espósito et al., 2005; Zhao et al., 2006). Moreover, new adult-generated neurons are able to play a significant role in hippocampal function, given that they receive functional afferents and generate excitatory post-synaptic potential (EPSP) in response to excitatory input from the perforant pathway and also release glutamate onto post-synaptic target cells, contributing to the long-term potentiation at the neuron population level (Snyder et al., 2001; van Praag et al., 2002; Espósito et al., 2005; Ge et al., 2006; Laplagne et al., 2006, 2007; Toni et al., 2008; Wang et al., 2008; Garthe et al., 2009).

A functional role played by new neurons is confirmed by the reduced memory functions observed after ablation or altered timing of differentiation of new neurons (Shors et al., 2001, 2002; Snyder et al., 2005; Saxe et al., 2006, 2007; Farioli-Vecchioli et al., 2008) and, on the other hand, by the observations that learning (or physical exercise) stimulates neurogenesis and that an increased neurogenesis stimulates learning and pattern separation (Kempermann et al., 1997a, 1998; van Praag et al., 1999; Ambrogini et al., 2000; Döbrössy et al., 2003; Creer et al., 2010; Sahay et al., 2011).

However, new adult-born neurons show responses in their activity which differ from those of mature neurons, such as: (1) a decreased induction threshold for LTP in the range of 1–1.5 months of age (Wang et al., 2000; Snyder et al., 2001; Schmidt-Hieber et al., 2004; Saxe et al., 2006; Ge et al., 2007); (2) an enhanced membrane excitability that, although it may have an homeostatic function in compensating the reduced glutamatergic input, may also contribute to strengthen the integration of young neurons in existing networks (Schmidt-Hieber et al., 2004; Mongiat et al., 2009); young neurons present the same overall firing behavior as mature neurons but they show longer spike latency (Mongiat et al., 2009), which may result in a different firing pattern, shown to be critical in the establishment of disambiguation of the input from the enthorinal cortex (pattern separation; Leutgeb et al., 2007); (3) in newborn neurons GABA, the major inhibitory neurotransmitter in the adult brain, exerts a tonic excitatory action owing to their high cytoplasmic chloride ion content (Ge et al., 2006, 2007). Considering also that young neurons of the dentate gyrus appear to be able to express and release GABA only until they are three weeks old (Gutiérrez et al., 2003; Zhao et al., 2010), this suggests a different regulation of new neurons compared to old neurons, which may differently modulate their activity.

Another interesting issue for the young dentate gyrus neurons concerns the expression of immediate early genes such as c-fos or Arc, which encodes an activity-regulated cytoskeletal-associated protein. The expression of immediate early genes is rapidly activated by learning tests and/or by the induction of LTP, and it has been proposed to be required to stabilize recent synaptic changes (Guzowski et al., 2005; Miyashita et al., 2008). In fact, c-fos or Arc are induced in dentate gyrus neurons following spatial or contextual memory training, and this occurs in correspondence to the recruitment of neurons into spatial memory circuits (Guzowski et al., 2005; Kee et al., 2007; Tashiro et al., 2007). Activated IEG expression matches in percentage the number of neurons electrophysiologically active (Guzowski et al., 2006), thus suggesting a direct correlation between firing activity and IEG activation. Notably, the expression of c-fos or Arc occurs in new neurons that have reached 4–8 weeks of age, but not earlier, indicating that only at this age new neurons give an enhanced contribution to behavior, being preferentially recruited into circuits supporting spatial memory, compared with existing granule cells (Kee et al., 2007; Farioli-Vecchioli et al., 2008).

As for the role played by young neurons in hippocampus-dependent cognition, recent experiments with specific protocols aimed to identify fine, rather than large, memory discriminations, have shown that new neurons are beneficial to pattern separation (Clelland et al., 2009; Sahay et al., 2011). These experiments have led to two theories; the first, by Aimone and Gage (Aimone et al., 2006, 2011), proposes that immature neurons, being more excitable, add information at low-specificity to mature neurons that are providing a highly specific but sparse representation of an event. In other words, new neurons would add resolution to the pattern separation which is normally exerted by the dentate gyrus architecture. In fact, young immature neurons would be able to encode more weakly the features of the environment, but would be very responsive to a wide range of inputs overlapping with one another. The high responsiveness of new neurons could reduce the need to have many of them to encode a large number of stimuli. Thus, this theory predicts that the utility of having more responsive immature neurons resides in two aspects: firstly, they would convey additional information to the sparse population of mature neurons that strongly encode a limited number of previously experienced features; secondly, as new neurons remain more excitable only for a limited period, once mature, they would also probably be more capable of responding to previously experienced inputs, thus maximizing the range of possible correlations between known inputs and new inputs. As a final result, new neurons would improve the resolution and correlation between new memories and known (older) memories of events, encoded by mature neurons. Specifically, a new neuron can be considered young, i.e., endowed with higher plasticity, until 4–8 weeks of age (Ge et al., 2007). The second theory, outlined by the Hen group (Sahay et al., 2011), is very similar, the essential difference being that while the first theory sees the new neurons as cell autonomous, individual encoding units, the second theory represents new neurons acting only as modulators of the sparse firing of mature dentate granule neurons.

It is worth noting that the evidence indicating the need of new neurons in the processes of learning and memory, either in experiments of pattern separation or previously, has essentially been obtained by acute reduction of the number of new neurons in mice models, by means of toxins, x-ray irradiation or virus-activated pro-drugs (Shors et al., 2001; Snyder et al., 2005; Saxe et al., 2007). The suppression of adult neurogenesis after these manipulations is not complete (Santarelli et al., 2003; Dupret et al., 2005) and learning may be sustained by the remaining adult-generated neurons or by the existing granule cells, or even by an alternative direct pathway from enthorinal cortex to CA3 (Aimone et al., 2006; Leutgeb et al., 2007).

Therefore, a functional prediction may be that an abrupt elimination of dentate gyrus cells, such as that performed in mouse models by genetically induced apoptosis or by toxins, may have a limited impact on cognitive function as their role may be vicariated. By contrast, a functional impairment of new dentate gyrus cells may crystallize memory circuits in a fixed state, and thus disrupting to a greater extent the cognitive function.

Thus, to study the function of newly generated neurons in the dentate gyrus we have adopted an approach different from ablation, by genetically modulating the differentiation and/or the rate of neurogenesis (i.e., the number of new neurons generated). Here, we will compare a model where only the rate of differentiation of adult-generated immature neurons changes - no difference occurring in the final number of mature neurons generated (transgene PC3/Tis21 driven by nestin [nestin/TgPC3/Tis21])—with two models presenting a change in the final number of mature neurons because of an impairment of either differentiation or survival of new neurons (knockout mice models of genes PC3/Tis21 and Btg1). Our results suggest that the rate of differentiation can be more critical for neuronal function than the absolute number of new neurons.

The actions of PC3/Tis21 and Btg1 on the development of neural progenitors are summarized in the following sections.

## Functional role of the PC3/Tis21 and Btg1 genes in neurogenesis

### Requirement of PC3/Tis21 for exit from the cell cycle and terminal differentiation of progenitor cells in the dentate gyrus and subventricular zone

PC3/Tis21 (also referred to as Btg2), a member of the BTG family of genes that comprises Btg1, Btg3, Btg4/PC3B, TOB, TOB2 (Bradbury et al., 1991; Fletcher et al., 1991; Rouault et al., 1992; Buanne et al., 2000; Matsuda et al., 2001; Tirone, 2001), is a transcriptional cofactor able to regulate and associate with the promoters of cyclin D1, RARβ, Id3, and Cxcl3 (Passeri et al., 2006; Farioli-Vecchioli et al., 2007, 2009, 2012a). PC3/Tis21 modulates transcription as a component of protein complexes. These can contain histone modifying factors such as the methyltransferase Prmt1 and the histone deacetylases HDAC4 or HDAC1, to which Tis21 is known to bind (Lin et al., 1996; Passeri et al., 2006; Farioli-Vecchioli et al., 2007), and/or transcriptional elements such as Caf1, CNOT8 or the transcription factor HoxB9 (Rouault et al., 1998; Prévôt et al., 2001, 2000).

A role for PC3/Tis21 in the proliferation and differentiation of neuroblasts was initially suggested by its expression in the developing neural tube and by its pattern of induction as an immediate early gene, activated at the very onset of the neural differentiation triggered by nerve growth factor (NGF) or fibroblast growth factor in PC12 cells (Bradbury et al., 1991). The neural character of this cell line was originally exploited to isolate the cDNA of PC3/Tis21, as PC12 cells originate from neurally committed progenitor cells of the neural crest that have migrated to the adrenal medulla; in the presence of NGF these cells rapidly stop proliferating and differentiate as orthosympathetic neurons (Rudkin et al., 1989). Eventually, it was shown that the expression of PC3/Tis21 was associated to proliferating neuroblasts undergoing a neurogenic division (Iacopetti et al., 1994, 1999). Indeed, in the neuronal PC12 cell line PC3/Tis21 is not able to trigger differentiation by itself, but only to synergize with NGF (Corrente et al., 2002; el-Ghissassi et al., 2002). In contrast, it was demonstrated *in vivo* that overexpression of PC3/Tis21 in a transgenic model is fully able to both inhibit the proliferation and induce the differentiation of progenitor cells, i.e., of neuroblasts of the neural tube during embryonic development, of granule precursors of cerebellum, and of adult progenitor cells in the dentate gyrus and in the SVZ (Canzoniere et al., 2004; Farioli-Vecchioli et al., 2007, 2008, 2009). PC3/Tis21 induces the proliferating progenitor cells to exit the cell cycle through direct repression of the cyclin D1 promoter and, with a probably independent action, to differentiate (Canzoniere et al., 2004; Farioli-Vecchioli et al., 2007). In fact, PC3/Tis21 also activates proneural genes through direct repression of the promoter of Id3, an inihibitor of proneural bHLH genes. The requirement of PC3/Tis21 for this dual action is particularly evident in hippocampal dentate gyrus progenitor cells, where the ablation of PC3/Tis21, besides accelerating their proliferation, leads to impairment of their terminal differentiation (Farioli-Vecchioli et al., 2009). In fact, stage 5 early post-mitotic dentate gyrus neurons lacking PC3/Tis21 are unable to terminally differentiate into stage 6, although they have already exited the cell cycle (Farioli-Vecchioli et al., 2009). Consistently, endogenous PC3/Tis21 is expressed in stage 5 neurons (Calretinin^+^) but not in stage 6 neurons (Calbindin^+^; Attardo et al., 2010). By chromatin immunoprecipitation, we have shown that the pro-differentiative action of PC3/Tis21 correlates with the inhibition of the promoter of the gene Id3, where PC3/Tis21 is recruited.

Overall, the functional profile of PC3/Tis21 appears that of a pan-neural regulatory switch of the transition from neural progenitor cell to early post-mitotic and then to mature neuron. The expression of PC3/Tis21 can be induced in response to several functional stimuli including learning, given that PC3/Tis21 is an immediate early gene highly induced in parallel with Arc and fos after memory tasks or in an enriched environment (Sirri et al., 2010; Vallès et al., 2011) and also in response to the Notch1/Delta pathway, given that PC3/Tis21 is induced by Delta1 (Hämmerle and Tejedor, 2007). It is worth noting that PC3/Tis21 is highly induced together with Arc and fos after somatosensory stimulation of a brain area which is not an adult neurogenic niche (the whisker-barrel cortex; Vallès et al., 2011); this suggests that PC3/Tis21 may be induced in response also to an acute depolarization and/or to a plasticity stimulus not directly related to neurogenesis, as previously observed (Bradbury et al., 1991; Qian et al., 1993).

Interestingly, ablation of PC3/Tis21 in the cerebellum causes a significant impairment of migration and differentiation of cerebellar precursors, but does not affect their proliferation; this further suggests that the anti-proliferative and pro-differentiative actions of PC3/Tis21 are dissociable, and also indicates that its anti-proliferative action in cerebellum may be vicariated by other related genes (Farioli-Vecchioli et al., 2012b).

Further studies will be useful to disentangle the PC3/Tis21-dependent activations of the differentiative and of the proliferative pathways during the maturation of neural progenitor cells, and to further clarify the action of PC3/Tis21 on proneural genes.

### Btg1 is required to maintain the pool of stem cells in the dentate gyrus and subventricular zone

Btg1, 65% homologous to PC3/Tis21, is also required for the control of the proliferation of progenitor cells in the dentate gyrus and SVZ, given that its ablation triggers *in vivo* a strong increase in the number of cycling progenitor cells early after birth (at P7), similar to that observed for PC3/Tis21 (Farioli-Vecchioli et al., 2012b). However, this early postnatal enhancement of proliferation is transient, since in the adult dentate gyrus and SVZ the pool of Btg1-null proliferating stem and progenitor cells strongly decreases, with a higher frequency of exit from the cell cycle followed within a few days by apoptosis (Farioli-Vecchioli et al., 2012b). Thus, while the ablation of Btg1 primarily causes a loss of control of cell cycle, accompanied by apoptosis (as normally occurs when a negative regulator of the cell cycle is suppressed; Lee et al., 1994), it appears that a second component takes place after ablation of Btg1, i.e., an age-dependent decrease of the proliferative capacity of progenitor cells. This latter is evident in the dentate gyrus progenitor cells—revealed by their increased exit from the cell cycle in adult Btg1-null mice—as well as in neural stem and progenitor cells isolated from SVZ of *Btg1*-null adult mice. In fact, primary SVZ neurospheres from these mice show a reduction of the ability to replicate by asymmetric division, responsible for self-renewal (Farioli-Vecchioli et al., 2012b). The frequency of apoptotic death and the deregulation of the cell cycle appear more marked in Btg1-null progenitor cells than in PC3/Tis21-null progenitor cells, suggesting that the control of proliferation exerted by Btg1 in progenitor cells of dentate gyrus and SVZ is more critical than that by PC3/Tis21.

The reduced number of Btg1-null adult-generated neurons of the dentate gyrus and of the olfactory bulb (i.e., the terminal migratory destination of the neurons generated in SVZ) is essentially the consequence of both apoptosis and loss of proliferative capacity of the pool of stem and progenitor cells, rather than of an impairment of differentiation. Hence, the functional profile of Btg1 differs from that of PC3/Tis21, whose main requirement, at least in the adult hippocampus, appears to be for terminal differentiation of stage 6 neurons (Farioli-Vecchioli et al., 2009). Overall, Btg1 appears to be required for the maintenance and self-renewal of stem cells in the adult dentate gyrus and SVZ, and its ablation presents a phenotype of loss of the stem cell pool similar to the knockout of p21 (Kippin et al., 2005) or of RBPj, effector of Notch (Imayoshi et al., 2010). However, while PC3/Tis21 is known to act in a pathway parallel to p21, being induced by p53 as is p21 (Rouault et al., 1996; Sionov et al., 2000), for Btg1 we only know that it is not induced by p53 (Cortes et al., 2000). It would be interesting to verify whether Btg1 is regulated by the Notch1/Delta pathway as is PC3/Tis21.

Notably, Btg1, unlike PC3/Tis21, is not acutely induced by memory training, suggesting that the chief function of Btg1 is to regulate the number of progenitor cells and neurons, rather than neural plasticity.

The role of Btg1 in cerebellar development has not yet been analyzed. However, Btg1 is co-expressed with Tis21 in the neuroepithelia of postnatal and adult cerebellum (Canzoniere et al., 2004; Farioli-Vecchioli et al., 2012b), making it reasonable to hypothesize that Btg1 may cooperate with the proliferative control exerted by PC3/Tis21 in the cerebellum.

## Correlating changes in differentiation and survival of dentate gyrus progenitor cells with hippocampus-dependent spatial and contextual memory

**Figure 1:**
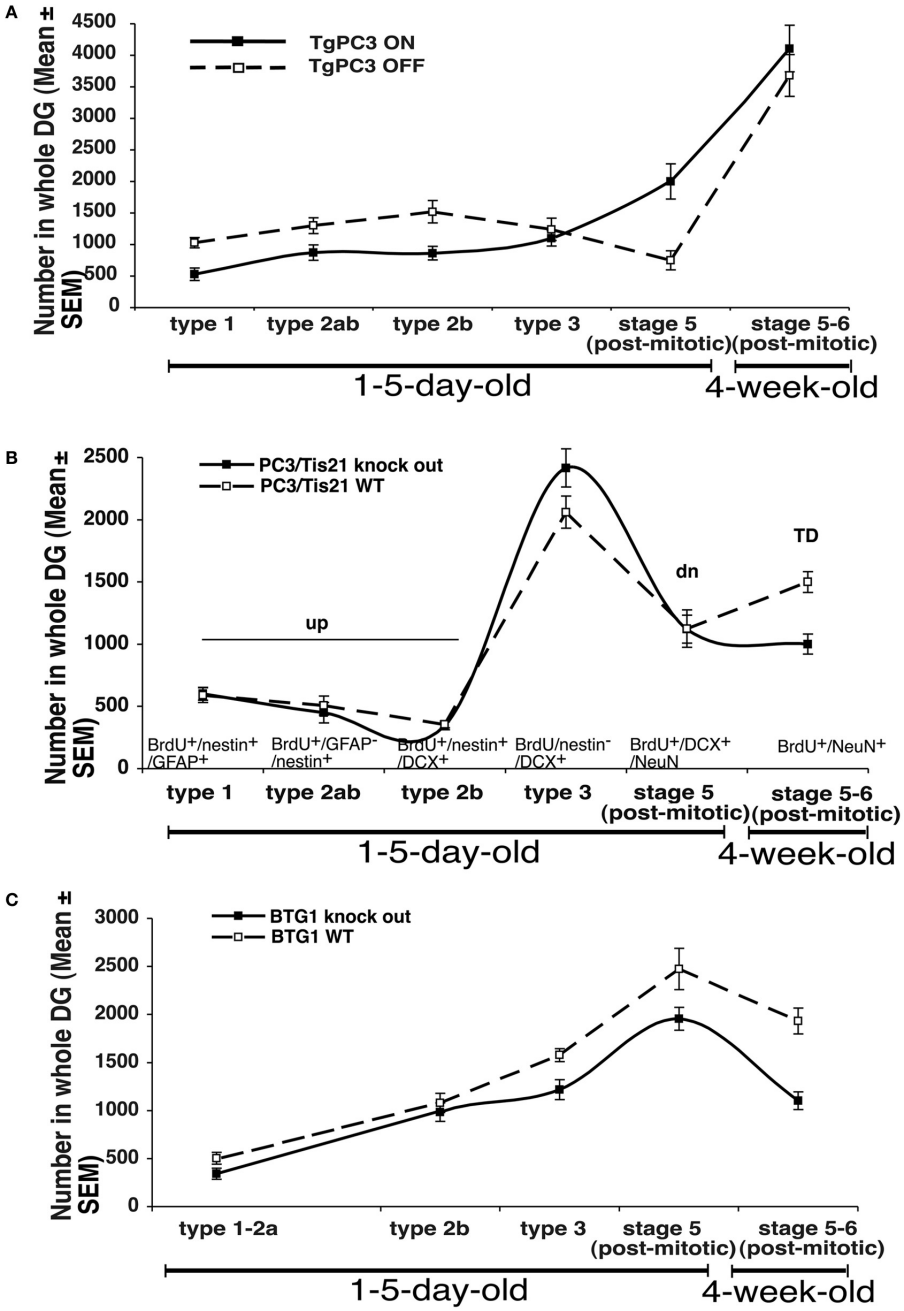
**Summary of the relative changes observed in the number of progenitor cell types and neurons of the dentate gyrus in the transgenic mouse model PC3/Tis21 (Tg PC3/Tis21) (A), or in PC3/Tis21 and Btg1 knockout mice (B,C)**. The markers used to identify the different types of neurons are indicated in panel **(B)**. The number of progenitor cells and neurons shown in the graph are obtained from data published in Farioli-Vecchioli et al. (2008) (Tg PC3/Tis21), (2009; 2012a; 2012b) (PC3/Tis21 and Btg1 knockout mice, respectively). TD: 28-day-old neurons BrdU^+^/NeuN^+^; td: 28-day-old neurons BrdU^+^/DCX^+^/NeuN^+^; dn: 1- to 5-day-old BrdU^+^/DCX^+^/NeuN^+^ progenitor cells; up: 1- to 5-day-old BrdU^+^/nestin^+^ progenitor cells.

In analyzing the role played by new neurons in the hippocampus-dependent memory, we aimed, as mentioned above, to compare the effects of the genetic modulation of the differentiation of new neurons with changes in their number. Clearly, the ablative approach does not allow one to monitor how new neurons are integrated into memory circuits, for instance by examining c-fos expression. Thus, we analyzed the data obtained after modulation of the differentiation and/or proliferation of the dentate gyrus progenitor cells by PC3/Tis21 and Btg1, and we attempted to correlate the cognitive function not only to the number of new neurons but also to ratios between dentate gyrus progenitor cells and neurons at different stages of differentiation. This analysis refers to three mouse models: (1) transgenic mouse PC3/Tis21 (Tg PC3/Tis21), where the overexpression of PC3/Tis21 in nestin-positive progenitor cells of the dentate gyrus induces 1- to 5-day-old neurons to differentiate faster without variation in the final number of mature 28-day-old neurons (Farioli-Vecchioli et al., 2008); (2) PC3/Tis21 knockout mice, where we observe a selective impairment in the differentiation of dentate gyrus mature 28-day-old stage 6 neurons (while the number of early postmitotic stage 5 neurons increases; Farioli-Vecchioli et al., 2009); (3) Btg1 knockout mice, where the final number of dentate gyrus adult-generated 28-day-old neurons decreases as a result of apoptosis and reduced replicative potential, without any evident change in the rate of differentiation, unlike in the PC3/Tis21 models (Farioli-Vecchioli et al., 2012b; Figures 1A–C).

Thus, to obtain a comprehensive measure of the state of neuron differentiation, we calculated the ratios between the number of each of the following cell types present in any mutant and in its corresponding wild-type mice, namely: i) young differentiated neurons (1- to 5-day-old BrdU^+^/DCX^+^/NeuN^+^ neurons, referred to as [*dn*]; Figures 1A–C and Table A1); (2) undifferentiated progenitor cells (1- to 5-day-old BrdU^+^/nestin^+^ progenitor cells referred to as [up]); (3) stage 6 mature 28-day-old neurons (BrdU^+^/NeuN^+^; [*TD*]); (4) stage 5 immature 28-day-old neurons (BrdU^+^/DCX^+^/NeuN^+^; [*td*]). Furthermore, dn/up, i.e., the ratio between *dn* and *up* values, is assumed to account for the presence of prematurely differentiated progenitor cells: if *dn*/up is higher than unity it should indicate an accelerated differentiation of new progenitor cells. On the other hand, the value *TD* should account for the actual number of new 4-week-old neurons potentially recruitable into memory circuits, while the value *td* represents the *TD* neurons at a preceding (transient) stage of maturation; therefore, an increase of the *td*/*TD* ratio should indicate an impairment of terminal differentiation (from stage 5 to 6), whereas a decrease may indicate a decrease of the mere number of new neurons generated.

Hence, an index of neurogenesis (IN)—proportional to the *dn*/up ratio and to the *td*/*TD* ratio—may reflect, when higher than unity, a situation of accelerated differentiation of progenitor cells and/or impaired differentiation (and function) of post-mitotic neurons. IN, if lower than unity, may reflect a situation where the decrease in the number of new neurons prevails on changes in differentiation ratios.

The following formula summarizes the above considerations:

(1) $$IN=\frac{dn}{up}\times \frac{td^{2}}{TD}$$

*td*^2^ is meant to emphasize impairment of terminal differentiation (*td* > 1) vs. a decrease of the number of stage 5 neurons (*td* < 1). One of the limits of equation (1) is its inability to distinguish between situations of decrease in the number of neurons (*td*) due, e.g., to apoptosis, and cases when terminal differentiation is accelerated (i.e., when the *td/TD* ratio decreases).

Table A1 shows the IN value for the three models analyzed: it is above unity when the early or terminal differentiation of neurons is altered with no change or small change in their number (Tg PC3/Tis21 and PC3/Tis21 knockout, respectively), and is smaller than unity when the mere number of new neurons is affected without apparent alteration of differentiation (Btg1 knockout).

**Figure 2:**
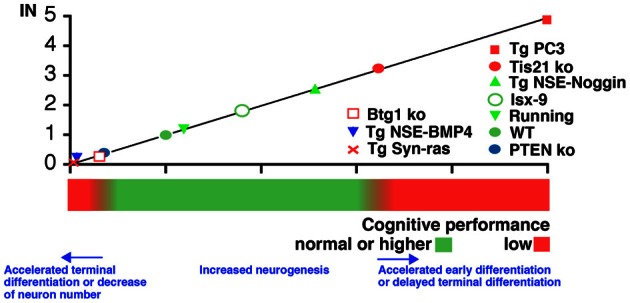
**Correlation between IN (neurogenesis index in the dentate gyrus) and hippocampus-dependent cognitive performance in situations of altered neurogenesis.** IN was calculated as described in the text for the conditions of altered neurogenesis observed in the mouse model Tg PC3/Tis21 (accelerated differentiation without change in neuron number), or in PC3/Tis21 and Btg1 knockout mice (impaired terminal differentiation, or reduced number of neurons generated, respectively). Additional mouse models were tested for their correlation with the proposed models: Tg NSE-BMP4 and Tg Syn-Ras presenting a strong decrease of the number of differentiated neurons; or Tg NSE-Noggin, Isx-9, or mice that underwent running exercise, showing an increase in the number of differentiated neurons; or PTEN knockout mice, presenting acceleration of terminally differentiated neurons. Assumptions in the calculation of IN for the additional mouse models were made (see Table A1) in order to use the data available in the respective publications (Gobeske et al., 2009; Manns et al., 2010; Amiri et al., 2012; Petrik et al., 2012).

If we correlate these three different situations to the memory performance we observe maximal impairment for Tg PC3/Tis21 (deep defect of spatial and contextual memory as well as of retrieval of acquired memories), intermediate for the PC3/Tis21 knockout (defect of contextual memory only), and minimal for Btg1 knockout mice (defect of pattern separation only) (see Figure 2).

To test whether IN can be used to predict memory performance, the IN of several other mouse models were obtained and correlated with the cognitive functions. Namely, we analyzed PTEN knockout where the terminal differentiation of neurons accelerates and the cognitive function is reduced (Amiri et al., 2012); or models associated to inhibition of neurogenesis, i.e., Tg Syn-Ras (Manns et al., 2010) and Tg NSE-BMP4 (Gobeske et al., 2009). The latter presented a full inhibition in the generation of new stem/progenitor cells (up), with also slightly accelerated differentiation from early to terminally differentiated neurons (*dn* and *td* are lower than *up* and *TD*, respectively). Tg NSE-BMP4 is accompanied by significant cognitive impairment, though not as evident as in Tg PC3/Tis21. Finally, we analyzed models of increased neurogenesis and cognitive function such as the Tg NSE-Noggin (Gobeske et al., 2009), Isx-9 (Petrik et al., 2012), or also voluntary running (Gobeske et al., 2009), where amplification of progenitor cells (up) increases and differentiation is also slightly unbalanced. A correlation between the IN index of the genes analyzed and the cognitive performance, forming a continuum between different degrees of maturation and/or generation of new neurons, appears to fit with the model proposed (Figure 2). At the flanks of the distribution are conditions where the unbalances in differentiation, or the decrease of new neurons generated, prevail. It should also be noted that in models where neurogenesis decreased (Tg NSE-BMP4, Tg Syn-ras, or Btg1 knockout), the proliferating stem cells were not abruptly eliminated by apoptosis but rather the continuous generation of stem cells and adult neurons was impaired; this plausibly may make a difference in terms of shaping the memory circuits, which could be more affected—depending on the extent of the decrease of new neurons generated—than by an acute ablation.

As a term of comparison, while IEG expression represents the functional endpoint of neuron activity, being the percentage of new neurons activated, IN aspires to enable a correlation between the whole dynamic of the process of maturation of new neurons and their function.

However, assumptions in the calculation of IN for the above test models were made (see Table A1), and certainly further tests will be required to check the IN in ranges greater than 3 (for highly accelerated differentiation). Moreover, in this computational approach aimed to take in account the process of differentiation, a further limit is the necessary caution to be exerted when considering genes that may change the identity of the adult born neuron and introduce new/different properties that would affect the cognitive function (though this does not seem the case for the genes analyzed here).

## Conclusions

Even without any formal calculation, from the mouse models analyzed it appears that alterations in parameters reflecting the process of differentiation alone, especially of young progenitor cells, have an impact on memory greater or equal than that seen when only the final number of neurons is drastically reduced. Further analyses will be necessary to substantiate this hypothesis. Nonetheless, this idea is perhaps not surprising, if we consider that the suppression by apoptosis of populations of newly born neurons has been shown to be functional to the hippocampus-dependent process of learning (Dupret et al., 2007; Kim et al., 2009). It has also been shown that the ablation of young neurons, since these are more excitable than mature granule cells, may reduce memory interference and increase discrimination in response to related but distinct stimuli, such as repetitive spatial representations (Saxe et al., 2007).

Two main reasons might underlie the high cognitive impact of an altered differentiation of new young neurons, namely: (1) the premature loss of the various functions described above peculiar to immature young neurons, which may result in faster aging (e.g., decrease of plasticity) of neurons and/or in the curtailing of an “instructive” developmental period (Tashiro et al., 2007); (2) a dominant negative effect. In fact, new neurons prematurely differentiated are unable to cooperate with the existing neurons, although they have not lost the basic LTP functions and can still, with time, recover their morphology and function (Farioli-Vecchioli et al., 2008); thus, the accumulation of “differently able” neurons in circuits creates a dominant negative effect in the whole circuitry. Consistent with this view, a dominant negative effect with marked impairment of hippocampal function appears to take place also when a prolonged survival of new neurons is enforced, in old Bax knockout mice, a condition which evidently impairs the plasticity within memory circuits, possibly by leading to saturation with less activated neurons (Kim et al., 2009).

On the other hand, the large reduction (more than 40%) observed in the number of new neurons in Btg1 knockout mice, but not in their differentiation, causes only a selective decrease of the finer memory discrimination. This agrees with the recently proposed role for new dentate gyrus neurons in pattern separation (Aimone et al., 2011; Sahay et al., 2011). However, it is possible that the role of new neurons in the memory circuitry, as gauged by their simple ablation, is underestimated, as suggested by the greater memory impairment occurring when the differentiation of the whole population of new neurons is altered. In that case, without change in the number of new neurons terminally differentiated, even the retrieval of already acquired memories is prevented (Farioli-Vecchioli et al., 2008).

A corollary of this would be that the architecture of circuits, unfolding from the interaction between existing and new neurons, can have a greater functional importance than the sheer number of new neurons, whose loss can evidently be compensated by alternative pathways and/or by the rewiring of circuits (see above).

Our observations may also concern the clinical evolution of human neurodegenerative diseases, such as the Alzheimer's disease, characterized by a progressively severe loss of hippocampus-dependent memory (Jacobs et al., 1995). This memory decrease has been associated not only to loss of neurons but also to impairment of the maturation and differentiation of neural progenitor cells (Lazarov and Demars, 2012; Mu and Gage, 2011). Thus, we can predict from our data that the correct timing of neuron differentiation is a variable that will have to be carefully considered in the study of neurodegenerative diseases, as well as in the development of efficient cell replacement therapies using neural stem cells (Selvaraj et al., 2012).

### Conflict of interest statement

The authors declare that the research was conducted in the absence of any commercial or financial relationships that could be construed as a potential conflict of interest.

## Appendix

**Table A1:** **Index of neurogenesis (IN), based on differentiation and/or number ratios of mutant vs. wild-type progenitor cells and neurons**.

TgPC3/Tis21 (Farioli-Vecchioli et al., 2008)—No change in the final number of mature differentiated neurons but the young differentiated neurons are more numerous. Strong impairment of spatial and contextual memory.			
IN = dn/up × td2/TD	4.8392973518		
	Tg PC3/WT	TgPC3	WT
dn: young differentiated neurons [active] ratio TgPC3 on vs. control (BrdU^+^ DCX^+^ NeuN^+^ 1–5-day-old)	2.6666666667	2000	750
up: young undifferentiated progenitor cells [inactive] ratio TgPC3 on vs. control (BrdU^+^ nestin^+^ 1–5-day-old)	0.5592783505	2170	3880
dn/up (a high ratio evidences an accelerated differentiation)	4.7680491551		
TD: terminally differentiated neurons, ratio TgPC3 on vs. control, (BrdU^+^ NeuN^+^ 28 gg)	1.038961039	4000	3850
td: early postmitotic neurons, ratio TgPC3 on vs. control (BrdU^+^DCX^+^NeuN^+^ 28 gg)	1.0268817204	191	186
td2/TD	1.0149428402		
PC3/Tis21 knockout (Farioli-Vecchioli et al., 2009)—The final number of mature differentiated neurons is reduced (because of impaired terminal differentiation) but the young differentiated neurons do not change. Impairment of contextual memory (spatial memory not affected).			
IN = dn/up × td2/TD	3.3400590464		
	Ko Tis21/WT	Ko Tis21	WT
dn: young differentiated neurons [active] ratio PC3/Tis21 ko vs. control (BrdU^+^DCX^+^NeuN^+^ 1–5-day-old)	0.9913793103	1150	1160
up: young undifferentiated progenitor cells [inactive] ratio PC3/Tis21 ko vs. control (BrdU^+^nestin^+^ 1–5-day-old)	0.9659863946	1420	1470
dn/up	1.0262870325		
TD: terminally differentiated neurons, ratio PC3/Tis21 ko vs. control, (BrdU^+^NeuN^+^ 28 gg)	0.687100894	1076	1566
td: early postmitotic neurons, ratio PC3/Tis21 ko vs. control (BrdU^+^DCX^+^NeuN^+^ 28 gg)	1.4953846154	486	325
td2/TD (a high ratio evidences an impairment of terminal differentiation)	3.2545076967		
Btg1 knockout (Farioli-Vecchioli et al., 2012a, b)—The final number of mature differentiated neurons is reduced (because of apoptosis) but the young differentiated neurons do not change. Mild cognitive impairment (pattern separation).			
IN = dn/up × td2/TD	0.2198125824		
	Ko BTG1/WT	Ko BTG1	WT
dn: young differentiated neurons [active] ratio Btg1 ko vs. control (BrdU^+^DCX^+^NeuN^+^ 1–5-day-old)	0.8125	1950	2400
up: young undifferentiated progenitor cells [inactive] ratio Btg1 ko vs. control (BrdU^+^nestin^+^ 1–5-day-old)	0.829	1326	1600
dn/up	0.9803921569		
TD: terminally differentiated neurons, ratio Btg1 ko vs. control, (BrdU^+^NeuN^+^ 28 gg)	0.5565085772	1103	1982
td: early postmitotic neurons, ratio Btg1 ko vs. control (BrdU^+^DCX^+^NeuN^+^ 28 gg)	0.3532338308	71	201
td2/TD (a low ratio evidences a reduced number of early post-mitotic neurons)	0.2242088341		
PTEN knockout (Amiri et al., 2012)—Increase of proliferation of progenitor cells accompanied by accelerated differentiation of terminally differentiated neurons (TD) vs. early post-mitotic neurons (td). Mild cognitive impairment (social behavior).			
IN' = dn/up × td2/TD	0.3373169225		
	PTEN ko/WT	PTEN ko	WT
dn: DCX^+^ 1 months	0.98	49	50
up: BrdU^+^Sox2^+^	0.9726775956	71.2	73.2
dn/up	1.0075280899		
TD: BrdU^+^NeuN^+^	4.9833333333	59.8	12
td: DCX^+^ 4 months	1.2916666667	62	48
td2/TD	0.334796544		
Syn-Ras Tg (Manns et al., 2010)—The final number of mature differentiated neurons and young differentiated neurons is reduced. Impaired spatial memory.			
IN' = dn/up × td2/TD	0.0382548368		
	Tg Ras/WT	Tg Ras	WT
dn: DCX^+^	0.1627906977	70	430
up: Brdu^+^/GFAP^+^	0.9444444444	8.5	9
dn/up	0.1723666211		
TD: BrdU^+^/Calb^+^	0.8275862069	48	58
td: BrdU^+^12dd	0.4285714286	300	700
td2/TD	0.2219387755		
Isx-9 (Petrik et al., 2012)—The final number of mature differentiated neurons and young differentiated neurons increases. Enhancement of spatial memory (no effect on fear conditioning).			
IN' = dn/up × td2/TD	1.8090219412		
	Isx-9/WT	Isx-9	WT
dn: BrdU^+^DCX^+^/BrdU^+^ (2 h BrdU)	1.8333333333	22	12
up: Brdu^+^GFAP^+^/BrdU^+^ (2 h BrdU)	1.25	5	4
dn/up (higher than unity: very slight acceleration of differentiation)	1.4666666667		
TD: BrdU+NeuN^+^/Brdu^+^ (30 days after BrdU)	1.4181818182	78	55
td: BrdU^+^DCX^+^/Brdu^+^ (30 days after BrdU)	1.3225806452	41	31
td2/TD	1.2334240508		
NSE-Noggin Tg (Gobeske et al., 2009)—Increase of neurogenesis, with a relative decrease of the number of young (dn) and mature differentiated (TD) neurons. Enhancement of spatial and contextual memory.			
IN' = dn/up × td2/TD	2.5103037429		
	Tg NSE-Noggin/WT	Tg NSE-BMP4	WT
dn: IdU^+^DCX^+^	2.6923076923	7	2.6
up: IdU^+^/Sox2^+^	3.7692307692	4.9	1.3
dn/up	0.7142857143		
TD: CldU^+^NeuN^+^	1.9565217391	2.25	1.15
td: CldU^+^DCX^+^	2.6222222222	11.8	4.5
td2/TD	3.5144252401		
NSE-BMP4 Tg (Gobeske et al., 2009)—Inhibition of neurogenesis, with decrease of both young (dn) and mature differentiated (TD) neurons Impaired spatial and contextual memory.			
IN' = dn/up × td2/TD	0.105405911		
	Tg NSE-BMP4/WT	Tg NSE-BMP4	WT
dn: IdU^+^DCX^+^	0.0961538462	0.25	2.6
up: IdU^+^/Sox2^+^	0.1153846154	0.15	1.3
dn/up	0.8333333333		
TD: CldU^+^NeuN^+^	0.1913043478	0.22	1.15
td: CldU^+^DCX^+^	0.1555555556	0.7	4.5
td2/TD	0.1264870932		
Running (Gobeske et al., 2009)—Increase of neurogenesis, with a decrease of the number of young (dn) neurons relative to progenitor cells (up) (as in NSE-Noggin). Enhancement of spatial memory.			
IN' = dn/up × td2/TD	1.2588937412		
	Running/WT	RUN	WT
dn: IdU^+^DCX^+^	2.1153846154	5.5	2.6
up: IdU^+^/Sox2^+^	2.9230769231	3.8	1.3
dn/up	0.7236842105		
TD: CldU^+^NeuN^+^	2.0173913043	2.32	1.15
td: CldU^+^DCX^+^	1.8733333333	8.43	4.5
td2/TD	1.7395622605		

The data used are published in Farioli-Vecchioli et al. (2008, 2009, 2012a, b). TD: 28-day-old neurons BrdU^+^/NeuN^+^; td: 28-day-old neurons.

BrdU^+^/DCX^+^/NeuN^+^; dn: 1- to 5-day-old BrdU^+^/DCX^+^/NeuN^+^ progenitor cells; up: 1- to 5-day-old BrdU^+^/nestin^+^ progenitor cells.

In order to use the data available for the PTEN knockout, Tg Syn-Ras, Isx-9, Tg NSE-Noggin, Tg NSE-BMP4 and running models in their respective publications.

(Manns et al., 2010; Amiri et al., 2012; Petrik et al., 2012; Gobeske et al., 2009), some assumption was made in the calculation of IN (as indicated above).
